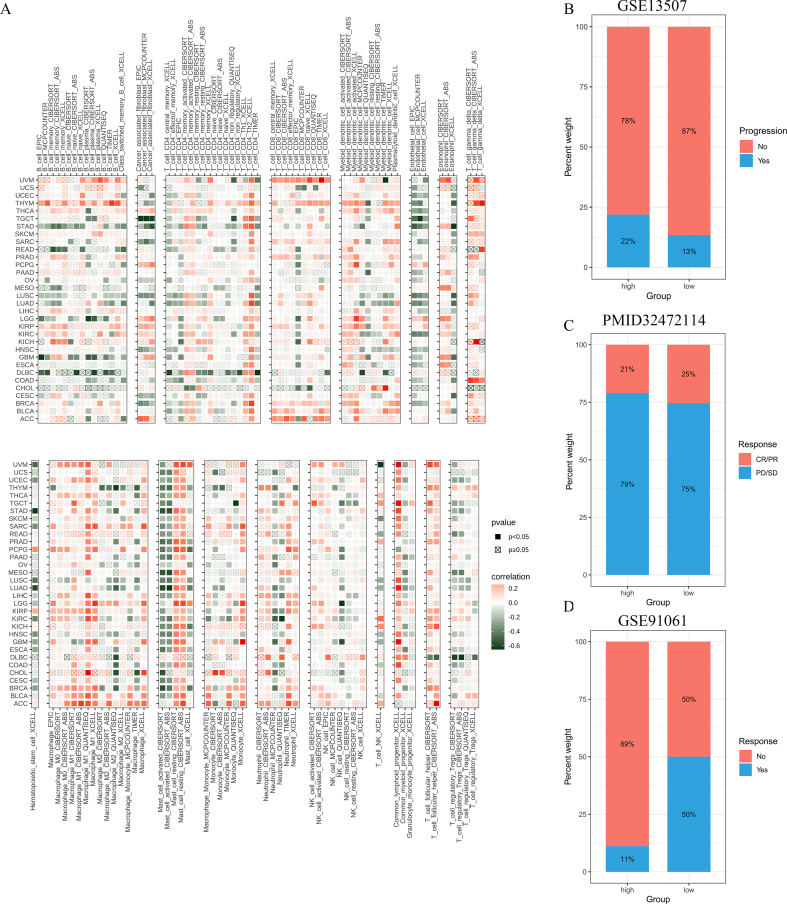# Integrated analyses reveal the prognostic and immunotherapeutic value of endoplasmic reticulum stress-related genes in cancer

**DOI:** 10.1016/j.gendis.2023.101187

**Published:** 2023-12-02

**Authors:** Qiuhua Lai, Lan Teng, Chengdong Liu, Yihong Lai, Jianxin Chen, Fangyao Wu, Mengduo Lei, Side Liu, Chaojun Zhu, Xiaohan Zhou

**Affiliations:** a Guangdong Provincial Key Laboratory of Gastroenterology, Department of Gastroenterology, Nanfang Hospital, Southern Medical University, Guangzhou, Guangdong 510515, China; b Department of Infectious Diseases, Nanfang Hospital, Southern Medical University, Guangzhou, Guangdong 510515, China; c Department of Gastroenterology, Zhuhai People's Hospital (Zhuhai Hospital Affiliated with Jinan University), Zhuhai, Guangdong 519050, China; d Department of Radiation Oncology, Nanfang Hospital, Southern Medical University, Guangzhou, Guangdong 510515, China

Endoplasmic reticulum (ER) stress is a procedure that results from increased protein release or improper ER protein folding, which is emerging as a possible driver of pathological conditions such as cancer, cardiometabolic diseases, rheumatic disease, and neurodegenerative diseases.^1^^,^^2^ Activating transcription factor 4 (ATF4), which is considered the primary controller of the cellular reaction when subjected to external stress,^3^ plays a vital role in amino acid metabolism, differentiation, metastasis, angiogenesis, and stress-related oxidative resistance.^4^ However, the prognosis value and immune signature of ATF4 activating genes in tumors are still unclear. Therefore, a better understanding of the role of ATF4 activating genes will promote new approaches to tumor treatment. In this work, we set up a model based on the expression level of ATF4 signaling-related genes, and ATF4 signaling score, which reflects the level of ER stress. Then we explored the expression profile of genes relevant to ATF4 signaling and evaluated the association between ATF4 signaling score and the prognosis of cancer patients. Additionally, our study explored the connection between ATF4 signaling score and tumor immunologic characteristics. Therefore, the current work provides a thorough analysis of the expression of 27 ATF4 signaling-related genes in 33 different kinds of cancers. Our results further demonstrate the potential of ATF4 signaling in tumor development and immunotherapy.

Using the GSEA dataset, we found 27 ATF4 activating genes that respond to ER stress (Table S1). Protein–protein association networks of 27 ATF4 activating genes proclaim close correlations between these genes (Fig. S1A). To investigate the characteristics of these genes, we assessed the overall mutation of ATF4 activating genes in pan-cancer. The waterfall plot revealed that the total mutation frequency of each gene was less than 1% and most mutations were missense mutations (Fig. S1B). The alteration patterns were different in various cancer types (Fig. 1A). We further examined the relationships between ATF4 activating genes (Fig. S2A). It showed that these genes had strong and positive correlations.

**Figure 1:**
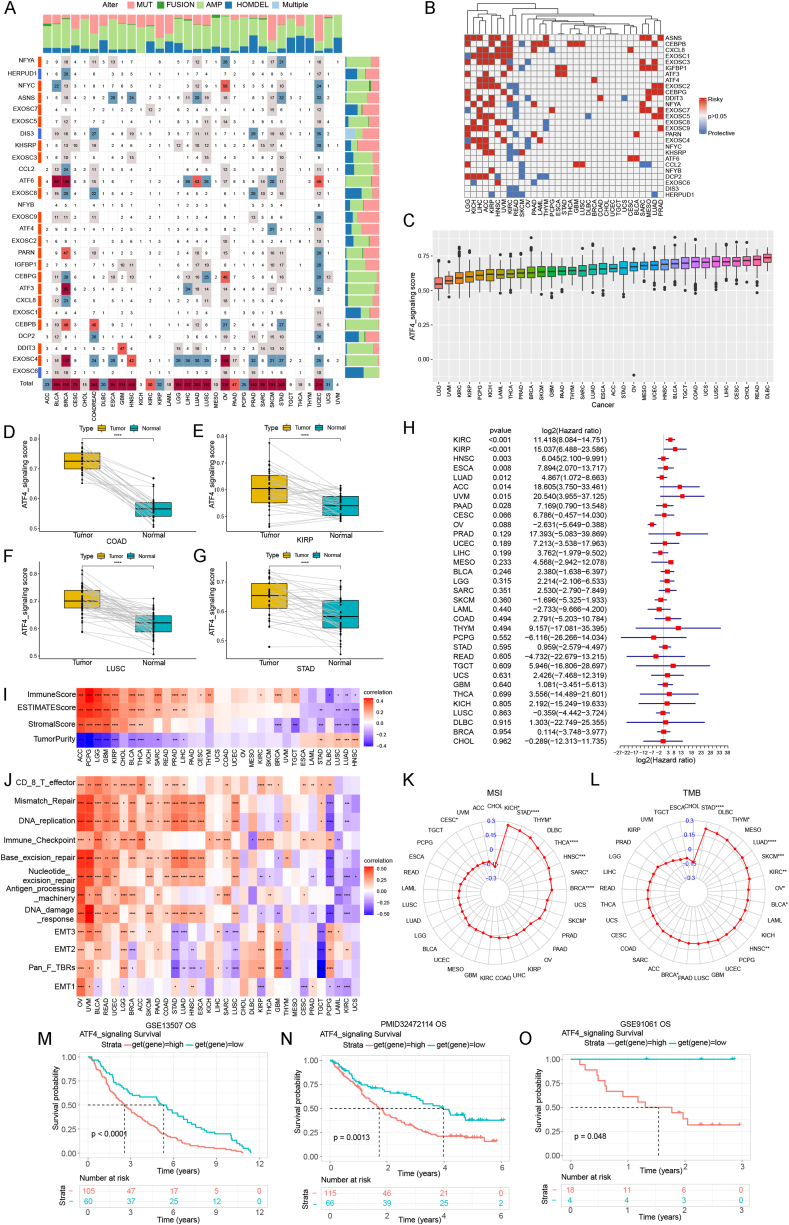
ATF4 activating genes are crucial in the prognosis and immunotherapy response of pan-cancer. **(A)** Profile of genetic alteration ATF4 activating genes in each cancer. **(B)** Univariate Cox analysis results of ATF4 activating genes in pan-cancer. **(C)** ATF4 signaling score in each tumor. **(D**–**G)** Tumor tissues have elevated ATF4 signaling scores compared with corresponding adjacent normal tissues in COAD, KIRP, LUSC, and STAD. **(H)** Forest plot implied the univariate Cox regression results for overall survival (OS). **(I)** Correlations between ATF4 signaling score and ImmuneScore, ESTIMATEScore, StromalScore, and TumorPurity. **(J)** Correlations between ATF4 signaling score and tumor microenvironment (TME)–related pathways. **(K, L)** Correlations between ATF4 signaling score and microsatellite instability (MSI) and tumor mutation burden (TMB). **(M**–**O)** The Kaplan–Meier OS analysis of ATF4 signaling score in GSE13507 (M), PMID32472114 (N), and GSE91061 (O) cohorts. ^∗^*P* < 0.05; ^∗∗^*P* < 0.01; ^∗∗∗^*P* < 0.001; ^∗∗∗∗^*P* < 0.0001.

As can be seen from Figure S2B, the expression of all the ATF4 activating genes was generally differentially expressed in pan-cancer. Additionally, we conducted univariate Cox regression analysis on each gene in the 33 cancers (Fig. 1B). The findings suggested that the majority of ATF4 activating genes were risk factors in tumors. We also calculated the risk scores and the result revealed that the majority of the genes were unfavorable for patient outcomes (Fig. S2C). These results indicate that ATF4 activating genes may contribute to cancer progression and correlate with the clinical outcomes of patients with many cancer types.

To comprehend the biological functions of ER stress related to the initiation of tumors, we established an estimated model of ER stress level based on the enrichment of ATF4 activating genes with the ssGSEA method. DLBC had the highest scores while LGG received the lowest (Fig. 1C). In contrast with normal tissues, the ATF4 signaling scores were significantly higher in multiple tumor tissues, such as BRCA, COAD, ESCA, HNSC, KIRC, KIRP, LUAD, LUSC, and STAD (Fig. 1D–G; Fig. S3A–E), while the score was decreased in PRAD (Fig. S3F). The scores varied across clinical stages in ACC, BLCA, ESCA, HNSC, KIRC, KIRP, LUAD, UVM, and OV. The ATF4 signaling scores exhibited a rising trend with the increase of clinical stage in the above cancers except OV (Fig. S4A–I).

We further assess the relationships between ATF4 signaling scores and four prognostic indexes. For overall survival, ATF4 signaling score worked as a danger factor in KIRC, KIRP, HNSC, RSCA, LUAD, ACC, UVM, and PAAD (Fig. 1H). For disease-specific survival, ATF4 signaling score worked as a danger factor in KIRC, KIRP, LUAD, THYM, PAAD, ACC, HNSC, ESCA, UCEC, PRAD, and UVM (Fig. S5A). For progression-free interval, ATF4 signaling score was a risk factor in KIRC, KIRP, UVM, PAAD, KICH, and LUAD (Fig. S5B). There was no significant interconnection between scores and disease-free interval (Fig. S5C). Compared with the high-expressed group, the low-expressed group has longer disease-specific survival in pan-cancer (Fig. S6A–L). These results suggest that ATF4 signaling scores are closely related to patient outcomes.

We applied GSEA to evaluate the pathways in 33 tumor types from TCGA (Fig. S7). The ATF4 signaling score was detected to be positively associated with multiple pan-cancer malignant pathways, such as MYC targets, mTORC1, TNF-α signaling via NF-κB, DNA repair, hypoxia, and IL6/JAK/STAT3 signaling, while it was negatively associated with Notch and Wnt/β-catenin signaling. These pathways were involved in remodeling the tumor microenvironment and motivating tumor progression.

We investigated the correlation between ATF4 signaling and the immune tumor microenvironment. The findings revealed that the immune score, stromal score, and ESTIMATE score were all strongly correlated with the ATF4 signaling score (Fig. 1I). According to the results, immune-related pathways like CD8 T effector, mismatch repair, DNA replication, and immune checkpoint pathways had a significant association with the ATF4 signaling score (Fig. 1J).

Using data obtained from reported studies,^5^ correlation analyses showed that ATF4 signaling was related to the increasing number of activated mast cells and CD4^+^ memory T cells, as well as M1-like macrophages, whereas it had an inverse correlation with dormant mast cells and CD4^+^ memory T cells (Fig. S8A). Using the ImmuCellAI database, ATF4 signaling has a positive relationship with the infiltrating levels of exhausted T cells, Th1 cells, and macrophages, and has a negative relationship with CD4^+^ T cells, Th17 cells, B cells, and naïve CD4^+^ T cells (Fig. S8B). Additionally, we explored the associations between the level of ATF4 signaling score and MHC genes (Fig. S8C), chemokines (Fig. S8D), and their receptors (Fig. S8E). Based on the immune infiltration feature in the TIMER2 database, the ATF4 signaling score had an association with the activated immune tumor microenvironment in tumors (Fig. S9A).

We also examined the relationships between the ATF4 signaling score level and microsatellite instability and tumor mutation burden, which were proposed to be related to the prognosis for various tumors after receiving immunotherapy. It is shown that the ATF4 signaling score has an obviously positive association with microsatellite instability in KICH, STAD, THYM, THCA, HNSC, SARC, BRCA, and SKCM, while a negative association was observed in CESC (Fig. 1K). For tumor mutation burden, the ATF4 signaling score showed a confidently positive association in STAD, THYM, LUAD, SKCM, KIRC, OV, BLCA, HNSC, and BRCA (Fig. 1L).

We examined the impact of the ATF4 signaling score on prognosis with datasets including prior treatment data as well as immunotherapeutic information. High scores of ATF4 signaling were noted in progressing phases (Fig. S9B) and non-responsive patients (Fig. S9C, D). In addition, higher ATF4 signaling scores were associated with poor overall survival compared with lower scores in various cancers (Fig. 1M−O). These findings implied that ATF4 signaling could influence the effectiveness of immunotherapy in some cancers.

This study provides a comprehensive description of the expression alterations of ER stress-related genes and their prognostic value based on the enrichment of ATF4 activating genes in pan-cancer. Furthermore, we uncover the impact of ATF4 activating genes on immune features and their prognostic value in immunotherapeutic patient. Our comprehensive analysis highlights the role of ATF4 activating genes in tumor development and immunotherapy.

## Author contributions

X.Z., C.Z., and S.L. designed the study. Q.L. and L.T. analyzed the data and wrote the original draft. C.L. provided technical support and data verification. Y.L. and J.C. participated in the discussion and language editing. F.W. and M.L. revised the manuscript. All authors approved the submitted version of the manuscript.

## Conflict of interests

The authors have no conflict of interests to declare.

## Funding

This work was supported by the China Postdoctoral Science Foundation (No. 2021M691469, 2022M721526), the Guangdong Basic and Applied Basic Research Foundation (China) (No. 2020A1515110872, 2021A1515111220, 2022A1515110165), the Guangzhou Science and Technology Plan Project (Guangdong, China) (No. 202201011554), the National Natural Science Foundation of China (No. 82102925, 82303366), the Key-Area Research and Development Program of Guangdong Province, China (No. 2022B0303020003), the President Foundation of Nanfang Hospital, Southern Medical University (No. 2020C007) and the College Students’ Innovative Entrepreneurial Training Plan Program (No. S202312121115).

## Figures and Tables

**figs1:**
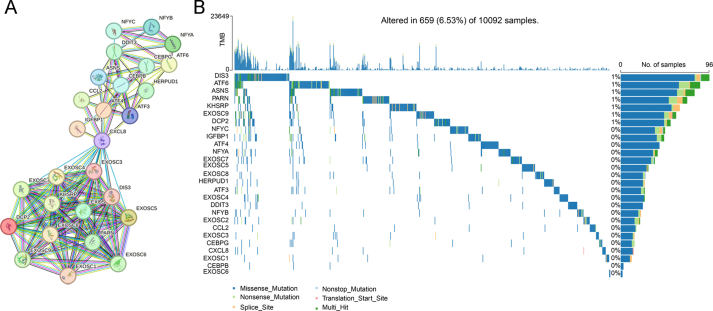


**figs2:**
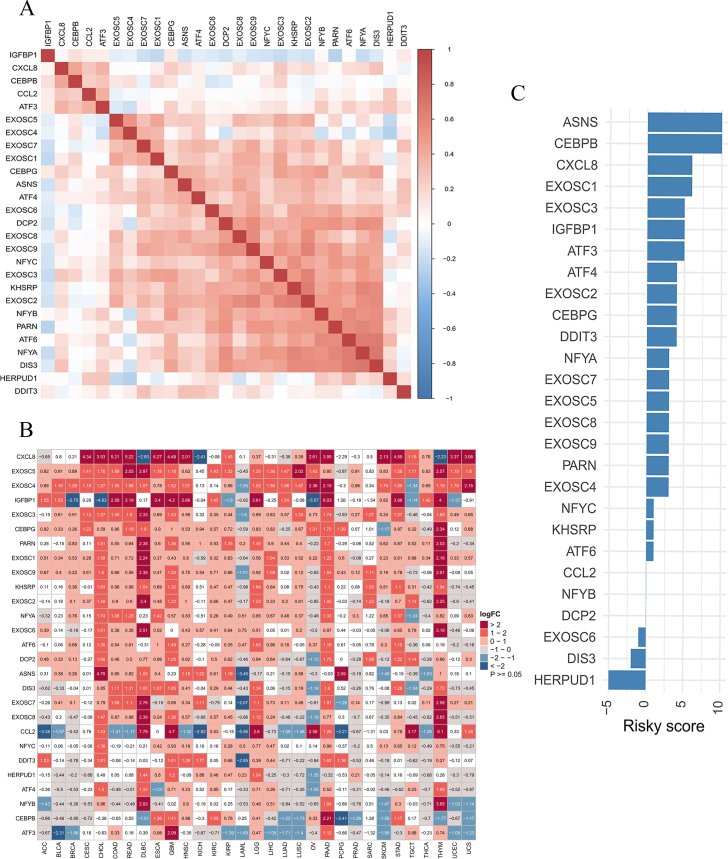


**figs3:**
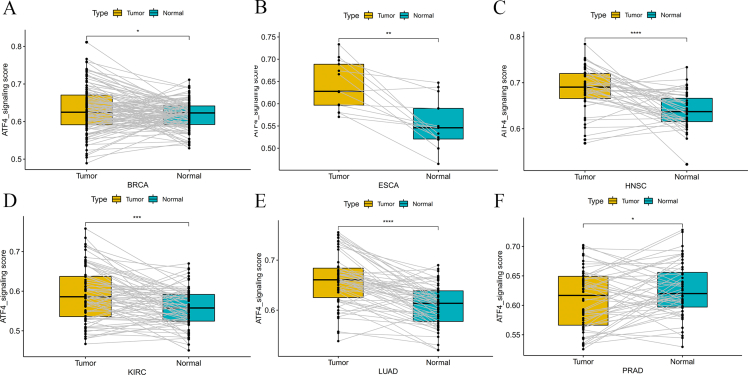


**figs4:**
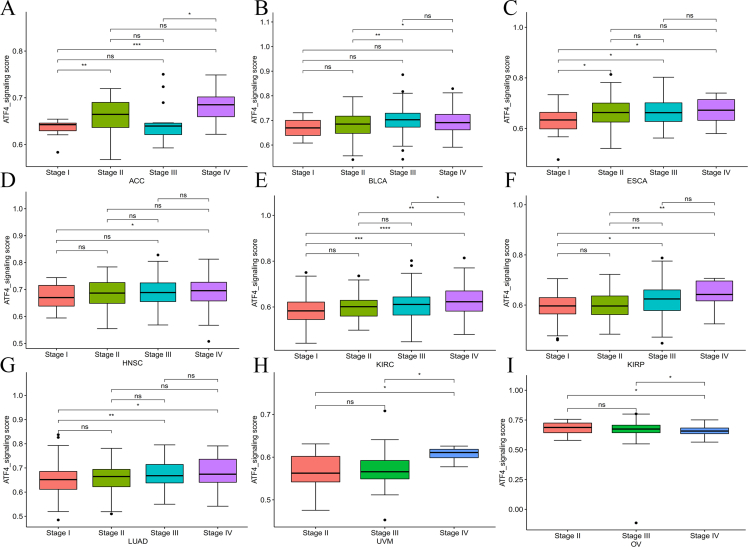


**figs5:**
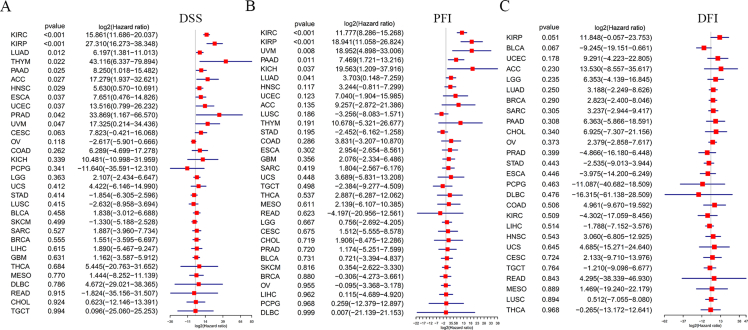


**figs6:**
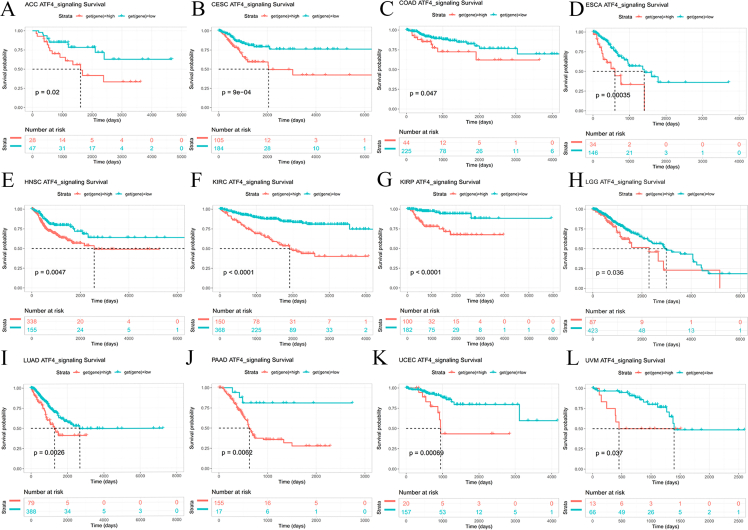


**figs7:**
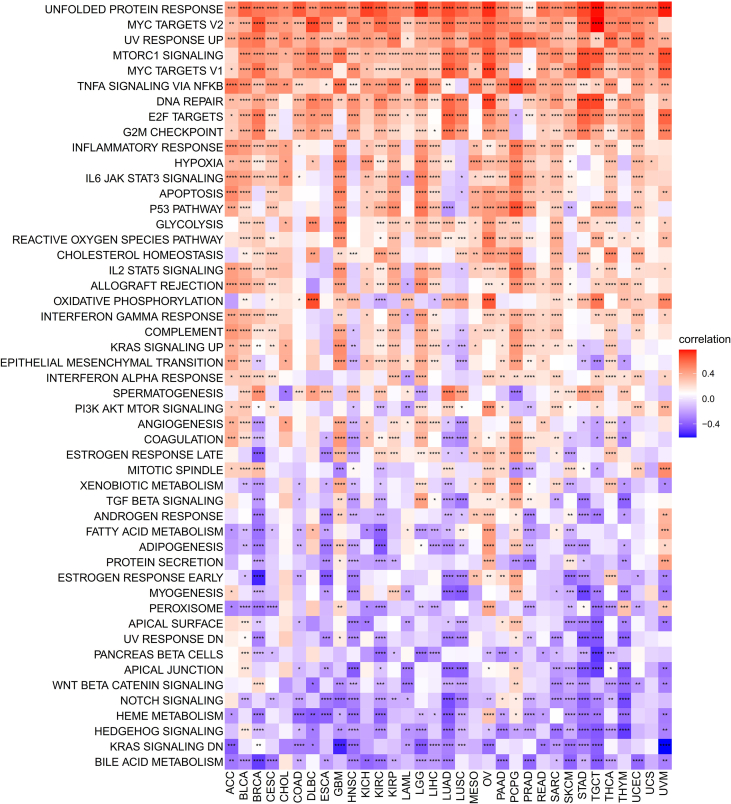


**figs8:**
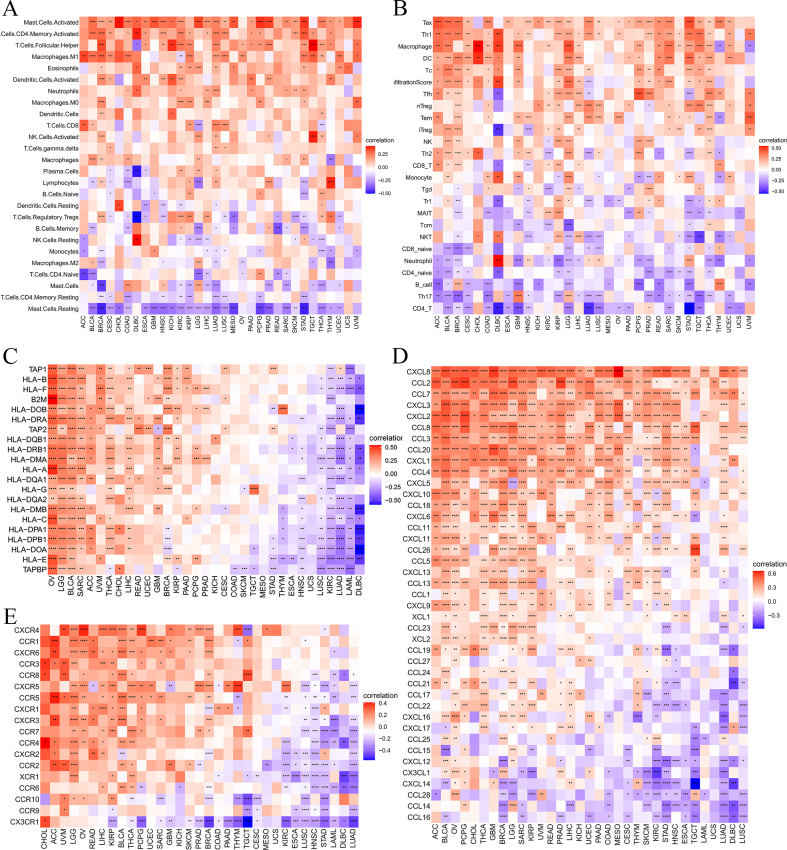


**figs9:**