# Supplementary material for: Integrated analyses reveal the prognostic and immunotherapeutic value of endoplasmic reticulum stress-related genes in cancer
Source: Genes Dis. 2023 Dec 2;11(6):101187. doi: 10.1016/j.gendis.2023.101187 (PMC11320448; doi:10.1016/j.gendis.2023.101187)
Supplement: Multimedia component 3 [file mmc3.docx]

**Methods**

**Data Collection**

A total of 27 ATF4 activating genes reacting to ER stress were identified from Gene Set Enrichment Analysis (GSEA) Molecular Signatures Database (https://www.gsea-msigdb.org/gsea/msigdb/index.jsp). The list of 27 genes is presented in Supplementary Table S1. The Cancer Genome Atlas (TCGA) and Genotype-Tissue Expression (GTEx) expression profiles and clinical data were gathered from the UCSC Xena database(<https://xenabrowser.net/datapages/>). The list of 33 cancer types is presented in Supplementary Table S2.

We accessed and examined the infiltration score of immune cells from the ImmuCellAI database (http://bioinfo.life.hust.edu.cn/web/ImmuCellAI/), TIMER2 database (<http://timer.cistrome.org/>) , and a recent study. The immunotherapy datasets we use were derived from the Gene Expression Omnibus database (<https://www.ncbi.nlm.nih.gov>).

**Mutation Analysis and Differentially Expressed Gene Analysis**

The cBioPortal database (<http://www.cbioportal.org/>) was utilized to collect the information about genetic mutation. The “maftool” R package was used to appraise the overall mutation of ATF4 activating genes in pan-cancer. The connection between ER stress-related genes was assessed using Pearson's correlation coefficient. Student’s *t*-test was utilized to calculate the difference of the 27 genes between carcinoma and normal tissues and the "ggplot2" R packages was used to visualize the result.

**ATF4 Signaling Score Analysis**

The ATF4 signaling score of individuals in the TCGA cohort was estimated using the single-sample gene set enrichment analysis (ssGSEA) which was implemented through “GSVA” package.

**Prognostic Analysis**

Using the R packages "survminer" and "survival", univariate regression analysis was applied to evaluate the impact of ATF4 signaling score on patient survival in pan-cancer, including overall survival (OS), disease-specific survival (DSS), disease-free interval (DFI), and progression-free interval (PFI) indicators. Additionally, Kaplan-Meier analysis was implied to plot the DSS curve based on ATF4 signaling score of patients in TCGA database.

**Gene Set Enrichment Analyses**

In order to investigate the biological capability that the ATF4 activating genes performed and their function across various tumors, gene set variation analysis (GSVA) with the R package "GSVA" was used to assess the relationship between ATF4 signaling score and 50 HALLMARK pathways relied on the GSEA Molecular Signatures Database (https://www.gsea-msigdb.org/gsea/msigdb/index.jsp)

**Tumor Microenvironment and Immune Cell Infiltration Analysis**

Stromal score, immune score along with tumor purity score for individuals in the TCGA were determined using the R package "ESTIMATE". The association of the ATF4 signaling score and the tumor microenvironment scores was analyzed. We inquired the association between ATF4 signaling score and infiltration of immunologic cell in pan-cancer. The "ggplot2" R package was utilized to visualize every heatmap in this part.

**Statistical Analyses**

Student’s *t*-test was utilized to assess differences across groups. The coefficient of Pearson correlation was applied in all analysis of correlation. R software 4.1.1 was applied to perform statistical analysis. P < 0.05 (two-tailed) was considered statistically significant.

**Supplementary figure legends**

**Figure S1.** Association and mutation characterization of ATF4 activating genes. (A) Protein–protein association networks of 27 ATF4 activating genes. (B) Mutation frequency of ATF4 activating genes in each tumor.

**Figure S2.** Aberrant expression of ATF4 activating genes in pan-cancer. (A) Correlation between ATF4 activating genes. The depth of the color represents the degree of the correlation. (B) Differential expressions of ATF4 activating genes in 31 tumors. FC: fold change. (C) Risky score reveals the patient prognostic risk for each gene in pan-cancer.

**Figure S3.** Differential analysis of ATF4 signaling score among tumor and normal samples. (A-E) Tumor tissues have elevated ATF4 signaling score compared to corresponding adjacent normal tissues in BRCA, ESCA, HNSC, KIRC, LUAD. (F) Tumor tissues have decreased ATF4 signaling score compared to corresponding adjacent normal tissues in PRAD.

**Figure S4.** Association between ATF4 signaling score and clinic stage in pan-cancer.

**Figure S5.** Correlations of ATF4 signaling score with survival in pan-cancer by forest plots. (A) Disease-specific survival (DSS); (B) Progression-free interval (PFI); (C) Disease-free interval (DFI).

**Figure S6.** The Kaplan-Meier DSS analysis of ATF4 signaling score.

**Figure S7.** Gene set variant analysis of ATF4 signaling score. Heatmap showed correlation between ATF4 signaling score and pathways.

**Figure S8.** Immune cell infiltration analysis. Correlation of ATF4 signaling score with immune cell subsets (A) and ImmuCellAI (B), MHC genes (C), chemokines (D) and chemokines receptors (E).

**Figure S9.** The association of ATF4 signaling with immune cell infiltration and immunotherapy response. (A) Immune cell infiltration analysis in TIMER2 database. (B-D) The percentage of progression and response in high-ATF4 and low-ATF4 signaling score groups in GSE13507, PMID32472114, and GSE91061 cohorts. CR: complete response; PR: partial response; PD: progressive disease; SD: stable disease. progression and response in

**Supplementary Table S1.** List of 27 ATF4 activating genes.

**Supplementary Table S2.** List of 33 types of cancer in TGCA Database.
