# Supplementary material for: Integrated analyses reveal the prognostic and immunotherapeutic value of endoplasmic reticulum stress-related genes in cancer
Source: Genes Dis. 2023 Dec 2;11(6):101187. doi: 10.1016/j.gendis.2023.101187 (PMC11320448; doi:10.1016/j.gendis.2023.101187)
Supplement: Multimedia component 4 [file mmc4.docx]

**Integrated Analyses Reveal the Prognostic and Immunotherapeutic Value of Endoplasmic Reticulum Stress-related Genes in Cancer**

**Background and Aim**

Endoplasmic reticulum (ER) stress is a procedure that results from increased protein release or improper ER protein folding, which is emerging as a possible driver of pathological conditions such as cancer, cardiometabolic diseases, rheumatic disease and neurodegenerative diseases. Activating transcription factor 4 (ATF4), which is considered as the primary controller of the cellular reaction when subjected to external stress, plays a vital role in amino acid metabolism, differentiation, metastasis, angiogenesis and stress-related oxidative resistance. However, the prognosis value and immune signature of ATF4 activating genes in tumors are still unclear. Therefore, better understanding the role of ATF4 activating genes will promote new approaches to tumor treatment.

In this work, we set up a model that based on the expression level of ATF4 signaling-related genes, ATF4 signaling score, which reflects the level of ER stress. Then we explored the expression profile of genes relevant to ATF4 signaling and evaluated the association between ATF4 signaling score with the prognosis of cancer patients. Additionally, our study explored the connection between ATF4 signaling score along with tumor immunologic characteristics. Therefore, the current work provides a thorough analysis of the expression of 27 ATF4 signaling-related genes in 33 different kinds of cancers. Our results further demonstrate the potential of ATF4 signaling in tumor development and immunotherapy.

**Key Findings**

**
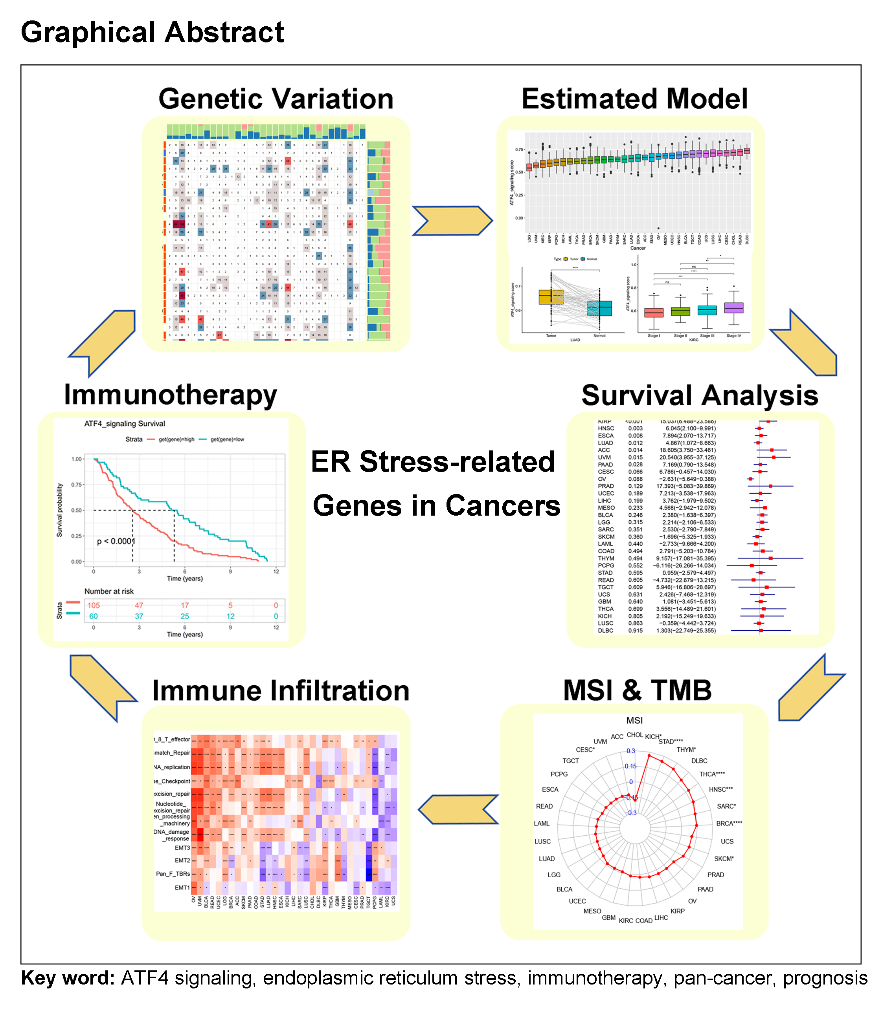
**

First, we found 27 ATF4 activating genes that response to ER stress in the GSEA dataset. To investigate the characteristics of these genes, we assess the overall mutation of ATF4 activating genes in pan-cancer. The waterfall plot revealed that the total mutation frequency of each gene is less than 1% and most mutations are missense mutations. The alteration patterns are different in various cancer types.

To explore the aberrant expression of ATF4 activating genes, we calculated the difference in these genes between tumor and normal tissues. The results showed that the expression of all the ATF4 activating genes were generally differentially expressed in pan-cancer. Additionally, we conducted univariate Cox regression analysis on each gene in the 33 cancers, which suggested that the majority of ATF4 activating genes were risk factors in tumors. We also calculated the risky scores and the result revealed that majority of the genes were unfavorable for patient outcomes. These results indicate that ATF4 activating genes may contribute to cancer progression and correlate with clinical outcomes of patients in many cancer types.

To comprehend the biological functions of ER stress related to the initiation of tumors, we established an estimated model of ER stress level based on enrichment of ATF4 activating genes with the ssGSEA method. Contrasting with normal tissues, the ATF4 signaling scores were significantly higher in multiple tumor tissues. The ATF4 signaling scores exhibited a rising trend with the increase of clinical stage in many types of cancer, while that showed a downward trend in OV. We further assessed the relationships between ATF4 signaling scores and four prognostic indexes. Compared to high-expressed group, low-expressed group had longer disease-specific survival in pan-cancer. These results suggested that ATF4 signaling scores are closely related to patient outcomes.

We then applied GSEA to evaluate the pathways in 33 tumor types from TCGA. The ATF4 signaling score was detected to be positively associated with multiple pan-cancer malignant pathways, such as MYC targets, mTORC1, TNF-α signaling via NF-κB, DNA repair, hypoxia, and IL6/JAK/STAT3 signaling, while it was negatively associated with Notch and Wnt/β-catenin signaling. These pathways were involved in remodeling the tumor microenvironment (TME) and motivating tumor progression.

Furthermore, we investigated the correlation between ATF4 signaling and the immune TME. The findings revealed that the immune score, stromal score, and ESTIMATE score were all strongly correlated with the ATF4 signaling score. According to results, immune-related pathways like CD8 T effector, mismatch repair, DNA replication, and immune checkpoint pathways had a significant association with the ATF4 signaling score. Using data obtained from reported studies, correlation analyses showed that ATF4 signaling was related to the increasing number of activated mast cells and CD4^+^ memory T cells, as well as M1-like macrophages, whereas it had an inverse correlation with dormant mast cells and CD4^+^ memory T cells. Using ImmuCellAI database, ATF4 signaling has a positive relationship with the infiltrating levels of exhausted T cells, Th1 cells and macrophages, and has a negative relationship with CD4^+^ T cells, Th17 cells, B cells and naïve CD4^+^ T cells. Based on immune infiltration feature in the TIMER2 database, the ATF4 signaling score had an association with activated immune TME in tumors.

In addition, we also examined the relationships between the ATF4 signaling score level and microsatellite instability (MSI) and the tumor mutation burden (TMB), which were proposed to be related to the prognosis for various tumors after receiving immunotherapy. It is shown that the ATF4 signaling score had an obviously positive association with MSI in KICH, STAD, THYM, THCA, HNSC, SARC, BRCA and SKCM, while negative association were observed in CESC. For TMB, the ATF4 signaling score showed a confidently positive association in STAD, THYM, LUAD, SKCM, KIRC, OV, BLCA, HNSC and BRCA.

Finally, we examined the impact of the ATF4 signaling score on prognosis with datasets including prior treatment data as well as immunotherapeutic information. High scores of ATF4 signaling were noted in progressing phases and non-responsive patients. In addition, higher ATF4 signaling scores were associated with poor OS compared with lower scores in various cancers. These findings implied that ATF4 signaling could influence the effectiveness of immunotherapy in some cancers.

**Significance of the Work**

This study provides a comprehensive description of the expression alterations of ER stress-related genes and its prognostic value based on enrichment of ATF4 activating genes in pan-cancer. Furthermore, we uncover the impact of ATF4 activating genes on immune features and their prognostic value in immunotherapeutic patient. Our comprehensive analysis highlights the role of ATF4 activating genes in tumor development and immunotherapy.

**Brief Introduction of the Team**

The team is from Nanfang Hospital of Southern Medical University, benefiting from the research platform of national key clinical specialties and Guangdong provincial key laboratory. The authors are mainly engaged in the molecular mechanism of tumor pathogenesis and metastasis. They have published dozens of articles on *Nature Communications*, *Cancer Communications*, *Oncogene*, *Journal of Experimental & Clinical Cancer Research*, *Acta Biomaterialia*, *Cancer Letters*, and so on.
